# An Exploration of the Tumor Microenvironment Identified a Novel Five-Gene Model for Predicting Outcomes in Bladder Cancer

**DOI:** 10.3389/fonc.2021.642527

**Published:** 2021-05-03

**Authors:** Xinjie Li, Jiahao Feng, Yazhou Sun, Xin Li

**Affiliations:** School of Medicine, Sun Yat-Sen University, Shenzhen, China

**Keywords:** bladder cancer, tumor microenvironment, prognosis, stroma, immune infiltration

## Abstract

Bladder cancer (BC) is one of the top ten most common cancer types globally, accounting for approximately 7% of all male malignancies. In the last few decades, cancer research has focused on identifying oncogenes and tumor suppressors. Recent studies have revealed that the interplay between tumor cells and the tumor microenvironment (TME) plays an important role in the initiation and development of cancer. However, the current knowledge regarding its effect on BC is scarce. This study aims to explore how the TME influences the development of BC. We focused on immune and stromal components, which represent the major components of TME. We found that the proportion of immune and stromal components within the TME was associated with the prognosis of BC. Furthermore, based on the scores of immune and stromal components, 811 TME-related differentially expressed genes were identified. Three subclasses with distinct biological features were divided based on these TME-genes. Finally, five prognostic genes were identified and used to develop a prognostic prediction model for BC patients based on TME-related genes. Additionally, we validated the prognostic value of the five-gene model using three independent cohorts. By further analyzing features based on the five-gene signature, higher CD8+ T cells, higher tumor mutational burden, and higher chemosensitivity were found in the low-risk group, which presented a better prognosis. In conclusion, our exploration comprehensively analyzed the TME and identified TME-related prognostic genes for BC, providing new insights into potential therapeutic targets.

## Introduction

Bladder cancer (BC) is a highly prevalent disease with an incidence of approximately 7% among all male malignancies and is the eighth most common cause of mortality (1). Although the incidence rate of BC has been decreasing, its death rate has not substantially reduced (2). The lack of understanding regarding the molecular mechanism of BC development results in the shortage of effective therapy for BC. Therefore, there is an urgent need to identify novel biomarkers for the early diagnosis of BC and to find effective methods to guide clinical treatments.

Tumor progression is a complicated process in which complex interactions occur between the tumor and the surrounding microenvironment. The surrounding microenvironment of tumor cells is referred to as the tumor microenvironment (TME), which includes immune cells, fibroblasts, and nearby stromal tissues (3, 4). The two major components of the TME are resident stromal cells and recruited immune cells (5, 6). Various studies have indicated the crucial role of stromal components and immune components in the TME on vascular invasion, adjacent tissue invasion, and drug resistance (7, 8). Thus, studying the heterogeneous components of the TME and their complex interactions is necessary to identify novel therapeutic targets.

Previous studies have screened some TME-related genes associated with survival outcomes, and few studies have systematically delineated the TME in BC and developed an accurate prognosis prediction model (9–12). Thus, it is imperative to fully use the clinical and biological information to conduct a more detailed analysis and to develop a robust and accurate prognostic prediction model of BC from the perspective of TME.

In this study, we calculated the scores of immune and stromal components of 406 BC patients from TCGA dataset and found significant correlations between these scores and prognosis. Patients were classified into three subtypes with distinct biological features based on the TME-related genes. Subsequently, a five-gene model based on TME-related genes was established and validated. The TME landscape of low- and high-risk BC groups predicted by our model was depicted, and immunotherapy factors such as tumor mutation burden (TMB), immune checkpoint, and chemosensitivity were explored. Based on this five-gene signature, we could predict the disease outcome and chemosensitivity that would be beneficial for further immunotherapy in BC.

## Materials and Methods

### Raw Data

Level 3 TCGA RNA-seq data of bladder cancer (including 19 normal and 411 tumor samples) and the corresponding clinical data were downloaded from TCGA dataset. By filtering out the normal paracancer tissue, the expression data for 411 cancer tissues were kept for downstream analysis, and 406 samples of which have available survival information. In addition, three separate bladder cancer cohort used in this study as validation datasets (GSE13507, GSE31684 and GSE32894) were downloaded from the GEO database (https://www.ncbi.nlm.nih.gov/geo).

### Generation of ImmuneScore, StromalScore and ESTIMATEScore

R package ‘estimate’ was used to calculate the ImmuneScore and StromalScore of tumor samples. Count matrix was normalized and log2 transformed. The higher ImmuneScore or StromalScore is, the larger amount of the immune or stromal components exits in TME. ESTIMATEScore is the sum of ImmuneScore and StromalScore denoting the overall proportion of immune and stromal components in TME.

### Consensus Clustering

Consensus clustering was introduced for classifying the BC patients into different subgroup. The K‐means algorithm with the Spearman distance was used for clustering. The cluster number was set to a range (1–10).

### Survival Curves

The relationship between kinds of scores and survival was explored by plotting the Kaplan–Meier curve using R package ‘survival’ and ‘survminer’. Log Rank test was used to test the differences of OS between defined high and low groups.

### Differential Gene Expression Analysis

Differential expression analysis was conducted using the R package ‘DESeq2’. The screening conditions for the differential genes were: |log2FoldChange| >1.5, padj <0.05. Heatmaps of differential genes were drawn using the R-package ‘pheatmap’. For subclass-specific genes, only genes with significant differences in expression (|log2FC| >1.5, padj <0.05) in all three possible comparisons were considered subclass-specific genes.

### GO and KEGG Enrichment Analysis and ssGSEA Analysis

GO and KEGG enrichment analyses were performed with the aid of R packages ‘clusterProfiler’, ‘enrichplot’, and ‘ggplot2’. Only terms with both *p*- and *q*-value of <0.05 were considered significantly enriched. ssGSEA analysis was conducted using R package ‘GSVA’. Hallmark geneset was downloaded from the Molecular Signatures Database (MSigDB).

### Tumor Infiltration Immune Cells (TICs) Profile

In combination with the LM22 signature matrix, normalized gene expression data (FPKM) were used to calculate the relative proportions of 22 types of infiltrating immune cells (encompass B cells, T cells, natural killer cells, dendritic cells, eosinophils, macrophages and neutrophils, among others.) *via* CIBERSORT algorithm. ImmuneAI (Immune Cell Abundance Identifier) computational method (http://bioinfolifehusteducn/ImmuCellAI#!/) was applied for predicting the immune checkpoint blockade response. Immune associated biomarkers for HLA, inflammation-promoting, MHC class I, Type I IFN Response were obtained from published paper (13). The enrichment scores were calculated using R package ‘GSVA’.

### Tumor Subtype Classification

Luminal biomarkers, basal biomarkers, squamous biomarkers, neuronal-differentiation biomarkers, and EMT-Claudin biomarkers, and the information of subtype of BC were achieved from the published study (14).

### Mutation Analysis

The mutation data of BC patients were obtained from the TCGA database. The mutation data of somatic variants were analyzed by using R package ‘maftools’.

### Establishment of the Five-Gene Risk Prediction Model

First, we assessed the relationships between the expression levels of the selected TME-related genes and overall survival of patients by univariate Cox regression analysis. The significant genes with *p* <0.05 were screened out for further analysis. We randomly divided the data from TCGA cohort as internal train set and internal validation set (1:1), the entire cohort as test cohort (n = 406). Subsequently, using R package ‘glmnet’, we performed the Least Absolute Shrinkage and Selector Operation (LASSO) analysis that could reduce the estimation variance while providing an explicable final model. We selected the *λ* with the least cross-validation error and identified the key genes affecting patients’ prognosis. Finally, multivariate Cox regression analysis was conducted to construct the TME-based signature for predicting the prognosis in BC patients. The time-dependent ROC curve performed by R package ‘survivalROC’ and Kaplan–Meier survival curve analysis performed by R package ‘survival’ were employed to verify the accuracy of the prognostic value of the five-gene signature.

### Prediction of Chemotherapy Responses and Candidate Small Molecules

The chemotherapy response for Methotrexate, Vinblastine, Doxorubicin, Cisplatin of each BC patient was calculated using R package ‘pRRophetic’ based on the Genomics of Drug Sensitivity in Cancer (GDSC) (https://www.cancerrxgene.org). To present potential drugs for five-gene signature risk group, the Connectivity map (CMap) (15) was performed to predict highly significant small molecule drugs for high-risk group compared to low-risk group.

### Statistical Analysis

All the statistical analyses were performed with R software and packages from the Bioconductor project. Wilcox-test was used to compare two groups with non‐normally distributed variables. For comparisons of three groups, Kruskal–Wallis test and cross Chi-Square-test were used as nonparametric methods. Pairwise comparisons were adjusted using ‘Holm’s method’. Univariate, multivariate Cox regression and Lasso regression were used for the selection of genes for the predictive gene signature. R package ‘glmnet’ was used for Lasso regression R packages ‘survivalROC’, ‘plotROC’ and ‘timeROC’ was used for the ROC curve analysis Area under the ROC curve (AUC) was used as an accuracy index to identify the predictive capacity.

## Results

### Immune and Stromal Scores Were Associated With the Prognosis and Progress of BC

**Figure 1:**
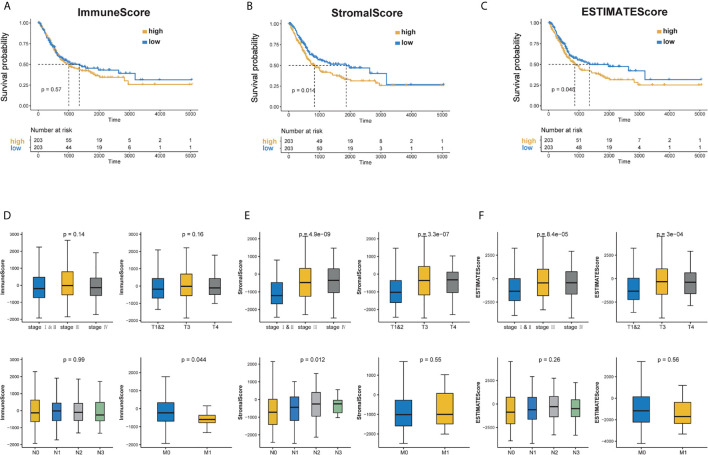
Correlation of scores with the survival of BC patients and clinicopathological staging characteristics. **(A–C)** Kaplan–Meier survival analysis for BC patients of high and low score in ImmuneScore, StromalScore and ESTIMATEScore. *p* = 0.57, 0.014 and 0.045 by Log Rank test. **(D)** Distribution of ImmuneScore in stage, T classification, N classification and M classification. The *p* = 0.14, 0.16, 0.99 and 0.044, respectively. **(E)** Distribution of StromalScore in stage, T classification, N classification and M classification. The *p* = 4.9e−09, 3.3e−07, 0.012, 0.55, respectively. **(F)** Distribution of ESTIMATEScore in stage, T classification, N classification and M classification. The *p* = 8.4e−05, 23e−04, 0.26 and 0.56, respectively.

To explore the correlation between the estimated proportion of immune and stromal components and the survival rate of BC patients, Kaplan–Meier survival analysis was performed for ImmuneScore, StromalScore, and ESTIMATEScore, respectively (16). Although ImmuneScore was not significantly correlated with the overall survival rate, StromalScore and ESTIMATEScore displayed a significant negative correlation with the overall survival (Log Rank test, *p <*0.05) (**Figures 1A–C**). These results suggest that the proportion of immune and stromal components could affect the survival of patients with BC.

Furthermore, clinical information was taken into account. The tumor node metastasis (TNM) staging system is the most widely used cancer staging system. As shown in **Figure 1D**, ImmuneScore was negatively correlated with M-stage (Wilcoxon signed-rank test, *p <*0.05). Patients in the low stages showed lower StromalScore and ESTIMATEScore scores (Kruskal–Wallis test, *p <*0.05) (**Figures 1E, F**). These results indicate that the proportion of immune and stromal components is associated with the prognosis and progression of BC.

### Identification of Differentially Expressed Genes (DEGs) Shared by ImmuneScore and StromalScore

**Figure 2:**
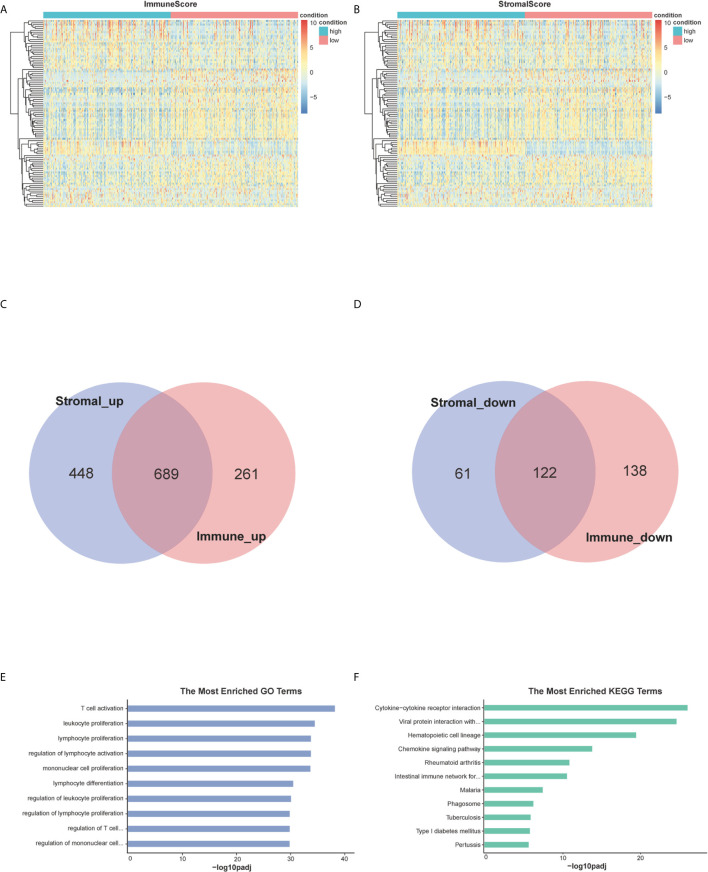
Heatmaps, Venn plots, and enrichment analysis of GO and KEGG for DEGs. **(A, B)** Heatmap for DEGs generated by comparison of the high score group vs. the low score group in StromalScore and ImmuneScore. Row is the gene, and column name is the samples which not shown in plot. Differentially expressed genes were determined by Wilcoxon rank sum test with *q <*0.05 and log2foldchange >1.5 as the significance threshold. **(C, D)** Venn plots showing common up-regulated and down-regulated DEGs shared by StromalScore and ImmuneScore. **(E, F)** GO and KEGG enrichment analysis for 811 DEGs, terms with *p* and *q <*0.05 were considered significant.

By analyzing expression data of all BC cases downloaded from TCGA database, we obtained the DEGs through the consolidation and analysis of different gene expression profiles between groups of high and low ImmuneScore and StromalScore. 1,210 DEGs were identified through the comparison between groups of high and low ImmuneScore. Among them, 950 genes were up-regulated and 260 genes were down-regulated compared to ImmuneScore group (|log2FC| >1.5, padj <0.05) (**Figure 2A**). Similarly, 1320 DEGs were identified between groups with high and low StromalScore, consisting of 1,137 up-regulated and 183 down-regulated genes (|log2FC| >1.5, padj <0.05) (**Figure 2B**). There are 689 up-regulated genes sharing by high score both in ImmuneScore and StromalScore (**Figure 2C**) and 122 down-regulated genes sharing by low scores (**Figure 2D**). These co-upregulated/downregulated 811 DEGs reflected the dynamic modulation of the immune and stromal components in TME and thus were possibly potential key factors for the status of TME. Functional enrichment analysis was performed based on the DEGs and results indicated that the DEGs are mainly mapped to the immune-related gene ontology (GO) terms, such as T-cell activation, leukocyte proliferation and lymphocyte proliferation (**Figure 2E**). Kyoto Encyclopedia of Genes and Genomes (KEGG) enrichment analysis showed the enrichment of cytokine-cytokine receptor interaction, chemokine signaling pathway, hematopoietic cell lineage and cell adhesion molecules (**Figure 2F**). These finds suggested that those DEGs were closely related to TME.

### Subtypes Divided Based on the TME-Related Genes

**Figure 3:**
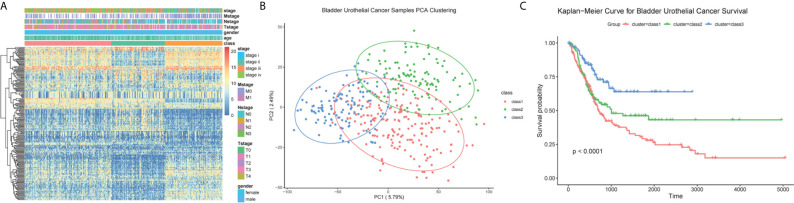
Classification of BC patients. **(A)** Heatmap for the expression of top 200 high variable genes. Clinical information was annotated for subclasses. **(B)** PCA plot for the assigned subclasses. **(C)** Kaplan–Meier survival analysis for BC patients grouped into three subclasses. Log Rank test comparing overall survival between the class1 and class3 subtypes reached a *p <*0.001. For the comparison between class 2 and class 3, *p* value <0.01. For class 1 and class 2, *p* = 0.067.

**Figure 4:**
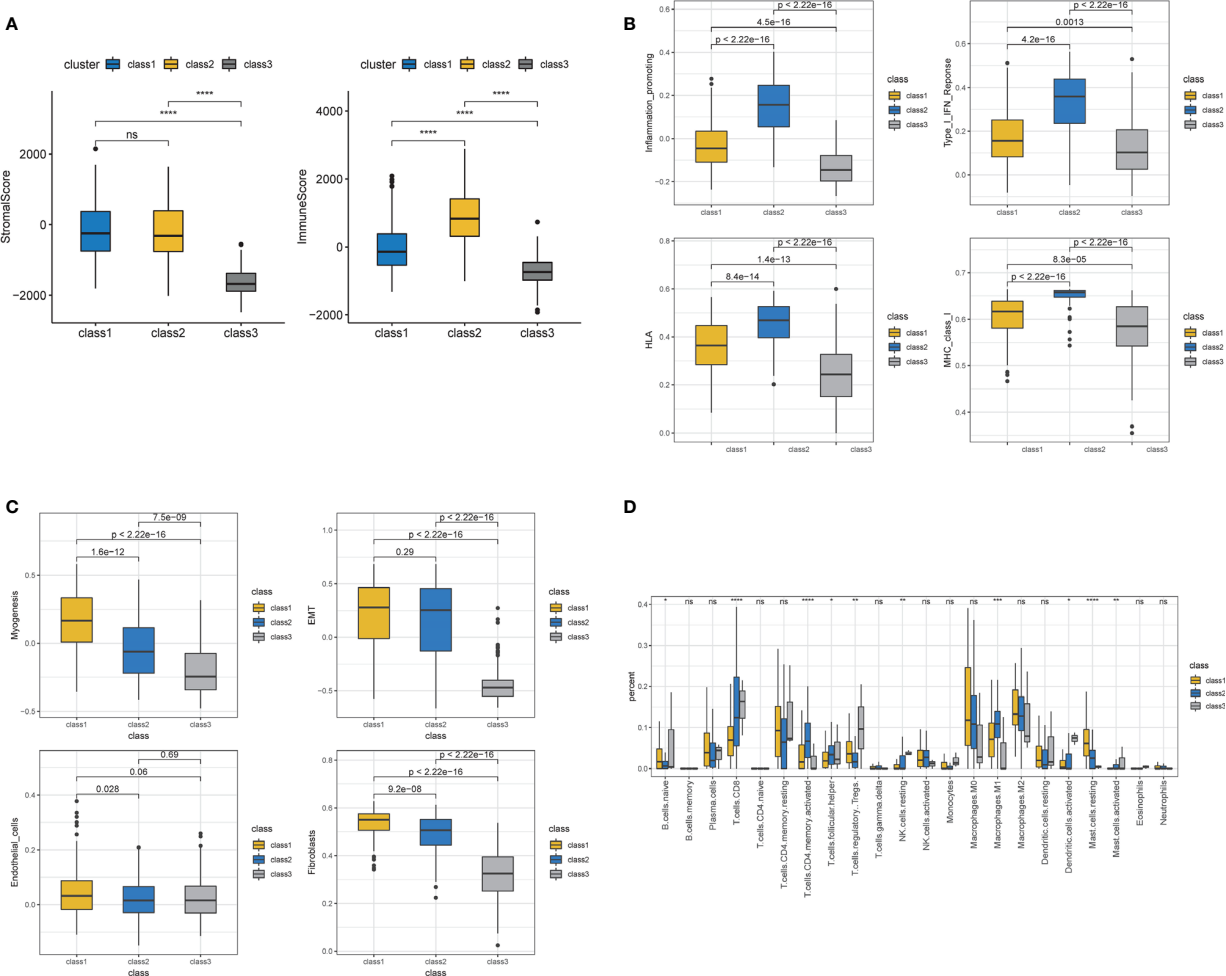
Immune and stromal features of subclasses. **(A)** Immune and stromal scores among three subclasses. **(B)** Immune-related molecular biomarkers among the three subclasses. **(C)** Stromal-related molecular biomarkers among three subclasses. **(D)** The fractions of 22 immune cells calculated by CIBERSORT among three subclasses.

Samples were then classified based on the 811 TME-related genes to explore the characteristics of the different subgroups in detail. Consensus clustering of the gene expression profiles yielded three subclasses (Classes 1–3), with 178, 110, and 118 cases in each subclass, which had significantly different expression patterns (**Figures S1A, B** and **Figure 3A**). The subclass assignments were concordant with the principal component analysis (PCA) results (**Figure 3B**). A significant prognostic difference was observed in these three subclasses (Log Rank test, *p <*0.0001), with the poorest overall survival for Class 1 patients and the best overall survival for Class 3 patients (**Figure 3C**). To better characterize the three subclasses, differential analyses of clinical features and biological features were performed. From the perspective of clinical data, patients assigned to the Class 1 subtype were relatively older and had more advanced disease, whereas the Class 3 subtype comprised patients with younger age and less advanced disease stages (Kruskal–Wallis test, *p <*0.05; Chi-Squared test, *p <*0.05) (**Table 1**). We observed that Class 1 and Class 2 both showed high scores in StromalScore and Class 2 also had significantly higher scores in ImmuneScore compared to the other two subclasses, whereas Class 3 showed significantly lower scores in both ImmuneScore and StromalScore compared to the other two subclasses (Kruskal–Wallis test, *p <*0.05; pairwise Wilcoxon signed-rank test, padj <0.05) (**Figure 4A**). The features of hallmarks and other typical biomarkers were compared among these subtypes, and there were remarkable differences among the three subtypes. Notably, Class 2 showed the highest enrichment scores in many immune signatures such as HLA, inflammation-promoting, MHC class I, and Type I IFN responses (Kruskal–Wallis test, *p <*0.05; pairwise Wilcoxon signed-rank test, padj <0.05) (**Figure 4B**). Class 1 and Class 2 both showed a high enrichment scores in stromal signatures such as myogenesis, endothelial cells, fibroblasts and EMT process, while class3 showed lowest enrichment scores in these signatures (Kruskal–Wallis test, *p <*0.05; pairwise Wilcoxon signed-rank test, padj <0.05) (**Figure 4C**). Other features which are not related to immune and stromal such as hypoxia, DNA repair, G2M checkpoint and MYC were significantly different among subclasses (Kruskal–Wallis test, *p <*0.05; pairwise Wilcoxon signed-rank test, padj <0.05) (**Figure S1C**). Expression of immune active markers like PRF1, GZMA, GZMB and immune suppressive markers like FOXP3, IL10, TGFB1 were screened in three subclasses (**Figure S1D**). Class 2 showed higher expression of these markers compared to the other two subclasses (Kruskal–Wallis test, *p <*0.05; pairwise Wilcoxon signed-rank test, padj <0.05). 22 kinds of immune cell fractions were then calculated using CIBERSORT algorithm, and significant differences were found among subclasses in CD8+ T cells, CD4+ activated memory cells, mast cells, M1 macrophages, Tregs and resting NK cells (Kruskal–Wallis test, *p <*0.05) (**Figure 4D**). We also checked the expression of the immune checkpoint genes and found that Class 2 showed higher expression of these genes compared to the other two subclasses (Kruskal–Wallis test, *p <*0.05) (**Figure S1E**). The response of patients to immune checkpoint blockade (ICB) therapy predicted by ImmuCellAI showed Class2 had the highest response compared to the other two subclasses (Chi-Squared test, *p <*0.05) (**Figure S1F**).

**Table 1:** Clinical Information of Subclasses.

	class1 (N = 178)	class2 (N = 110)	class3 (N = 118)	Overall (N = 406)
**Age (p <0.05)**				
Mean (SD)	70.7 (9.83)	67.5 (9.65)	63.9 (10.8)	67.9 (10.4)
Median [Min, Max]	71.0 [45.0, 89.0]	67.0 [43.0, 88.0]	65.5 [34.0, 84.0]	68.5 [34.0, 89.0]
**Gender(chi sq. p >0.05)**				
Female	54 (30.3%)	30 (27.3%)	22 (18.6%)	106 (26.1%)
Male	124 (69.7%)	80 (72.7%)	96 (81.4%)	300 (73.9%)
**Grade(chi sq. p <0.05)**				
High Grade	177 (99.4%)	109 (99.1%)	97 (82.2%)	383 (94.3%)
Low Grade	0(0%)	1 (0.9%)	19 (16.1%)	20 (4.9%)
**Stage(chi sq. p <0.05)**				
stage i	0(0%)	0(0%)	3 (2.5%)	3 (0.7%)
stage ii	25(14.0%)	37 (33.6%)	66 (55.9%)	128 (31.5%)
stage iii	67 (37.6%)	44 (40.0%)	30 (25.4%)	141 (34.7%)
stage iv	86 (48.3%)	29 (26.4%)	17(14.4%)	132 (32.5%)
**diagnosis subtype(chi sq. p <0.05)**				
Papillary	39 (22.3%)	21 (19.4%)	71 (60.2%)	131 (32.7%)
Not-papillary	136 (77.7%)	87 (80.6%)	47 (39.8%)	270 (67.3%)
**Origin(chi sq. p <0.05)**				
Bladder, NOS	92 (51.7%)	60 (54.5%)	80 (67.8%)	232 (57.1%)
Wall, dome, trigone, neck of bladder	86 (48.3%)	50 (45.5%)	38 (32.2%)	174 (42.9%)

**Figure 5:**
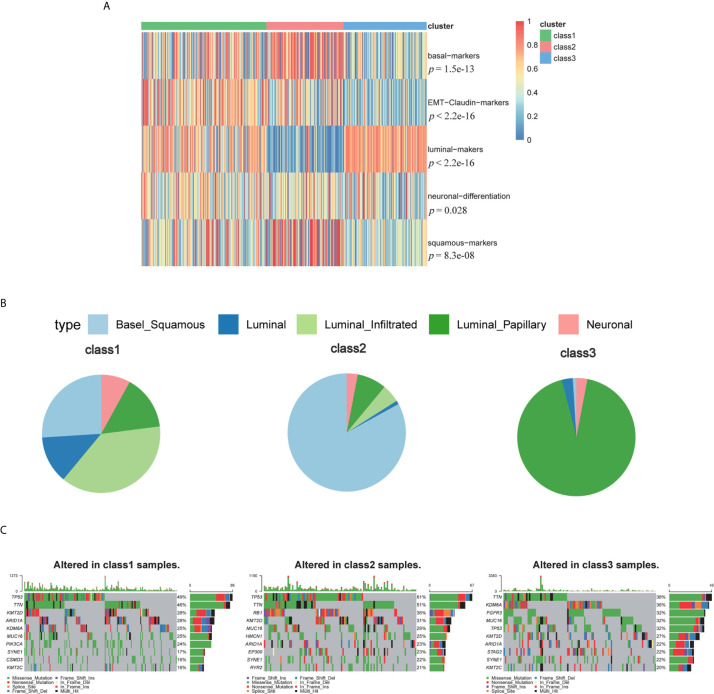
The biomarkers of other subtypes and mutations among three subclasses. **(A)** The biomarkers of other published subtypes among three subclasses. **(B)** Percent of Robertson’s subtypes. **(C)** Top mutations in class 1, class 2 and class 3.

Furthermore, we compared our classification with that of a previous study (**Figures 5A, B**). The previous classification identified five subtypes: luminal-papillary, luminal-infiltrated, luminal, basal-squamous, and neuronal (14). The neuronal subtype had the poorest overall survival time, whereas the luminal-papillary subtype had the best overall survival time. However, the basal-squamous subtypes had moderate overall survival time. In our study, Class 3, with the highest luminal marker expression and lowest basal and neuronal marker expression, demonstrated better survival times, which was similar to luminal-papillary subtype. Class 2 was similar to basal-squamous subtype. Class 1, the subclass with the poorest prognosis, showed a farraginous expression of markers and contained most of the neuronal samples. We also explored the genomic features of the three subtypes and found that Class 3 had a significantly lower mutation frequency of TP53 (32%) than that of Class 1 (49%) and Class 2 (61%), whereas Class 3 had a higher mutation frequency of FGFR3 (32%) and Class 2 had a higher mutation frequency of RB1(36%) (**Figure 5C** and **Figure S1G**). Thus, our TME-based classification identified three subtypes with significantly different clinical and biological features.

### Development and Validation of TME-Based Prognostic Model

**Figure 6:**
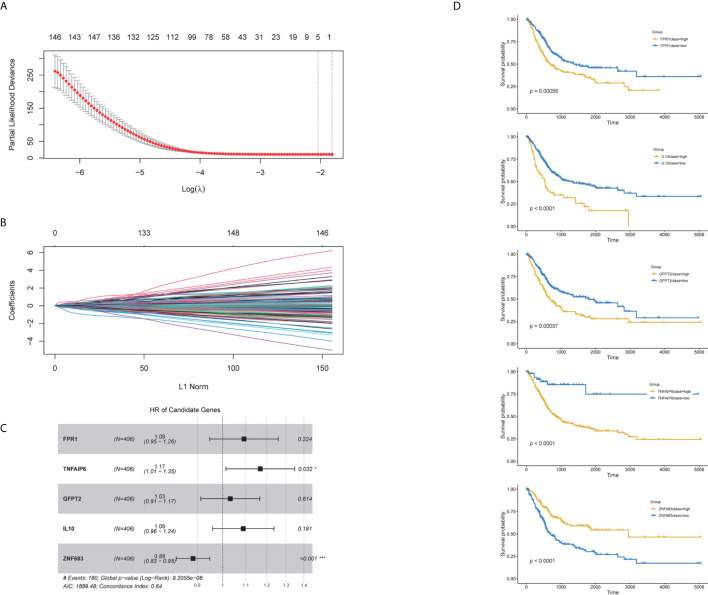
Characteristics of the prognostic gene signature. The prognostic index was imputed as follows: (0.234 ∗ FPR1) + (0.051 ∗ TNFAIP6) + (0.145 ∗ GFPT2) + (0.106 ∗ IL-10) + (−0.172 ∗ ZNF683). **(A)** Identification of the optimal penalization coefficient lambda in the Lasso regression model. **(B)** LASSO Cox regression algorithm was used to identify the most robust prognostic genes. **(C)** Forest plots presenting the multivariate Cox proportional hazards regression analysis of prognostic selected genes in overall survival**. (D)** Kaplan–Meier curves for patients grouped by expression levels of selected genes.

**Figure 7:**
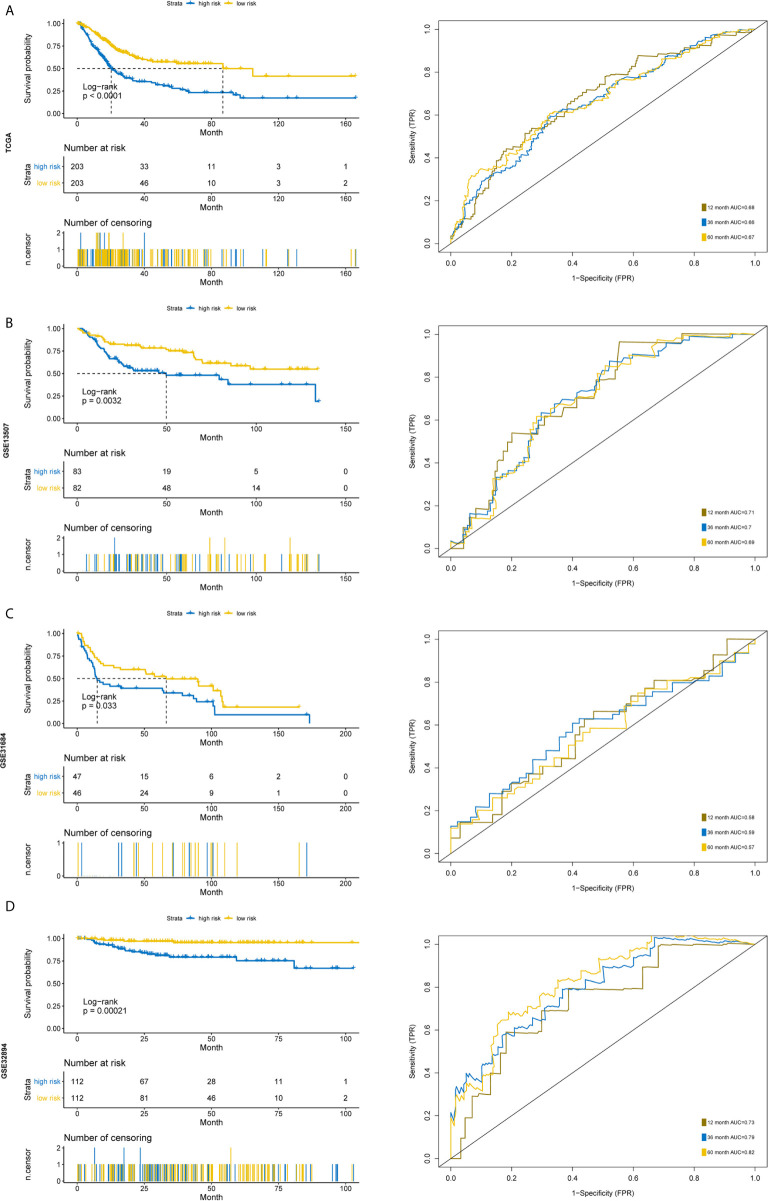
Time-dependent ROC analysis and Kaplan–Meier analysis for the validation of prognostic model in TCGA, GSE13507, GSE31684 and GSE32894. Kaplan–Meier curve and time-dependent ROC analysis of risk score in **(A)** entire TCGA cohort, **(B)** GSE13507 cohort, **(C)** GSE31684 cohort, **(D)** GSE32894 cohort.

Our above results suggested that the proportion of immune and stromal components can significantly influence the tumor progression and prognosis of BC, and TME-related genes could well classify the BC samples. Thus, to further explore the prognostic significance of the TME-related genes, univariate cox analysis was conducted to reduce the noise of gene without prognostic value (*p <*0.05 was considered to have prognostic value). The TCGA cohort was randomly divided into one train set and one validation set (1:1). Then Lasso regression was employed to present key genes for the establishment of a prognosis prediction model in internal train set (**Tables S1, S2** and **Figures 6A, B**). The five genes fitting into the model were FPR1, TNFAIP6, GFPT2, IL-10 and ZNF683 (**Figure 6C**). The K–M plot demonstrated that overexpression of FPR1, TNFAIP6, GFPT2, IL-10 and low expression of ZNF683 were associated with the poor overall survival of BC patients (**Figure 6D**). The prognostic value of five-gene signature was validated in the internal validation set and the entire TCGA cohort (**Figures S2A, B** and **Figure 7A**).

Furthermore, we used another three independent cohorts (GSE13507, GSE31684, GSE32894) to verify the prognostic value of our five-gene signature. The risk score for each sample was calculated using the model, and patients were divided into high- and low-risk groups according to the median of risk score. Log Rank test was used to test the differences of overall survival between the two groups. To evaluate the power of the prognostic model, time‐dependent Receiver Operating Characteristic (ROC) curves were employed to assess the sensitivity and specificity. The results indicated that the signature could accurately predict the overall survival of BC patients in all three cohorts (**Figures 7B, D**). The patients with higher risk scores had a significantly worse prognosis.

Since risk score has such a distinctive distinction, we further wonder whether the signature could become an independent predictor. Considering the age, stage information and risk score together in a multivariate model, the risk score based on five-gene model was still independent prognostic factor (**Figures S3A, B**). Moreover, this five-gene model had a higher concordance index score compared to the clinical stage model and higher area under curve (AUC) values for one- and three-year survival (**Figures S3C–F**). These results indicate that the five-gene signature is an accurate, robust and independent predictive tool for the prognostic prediction of BC patients.

### The Differences of Functional Annotation, Immune Cell Fraction, Mutation Profile, CNV, TMB, Sensitivity to Chemotherapy Among the High- and Low-Risk Groups

**Figure 8:**
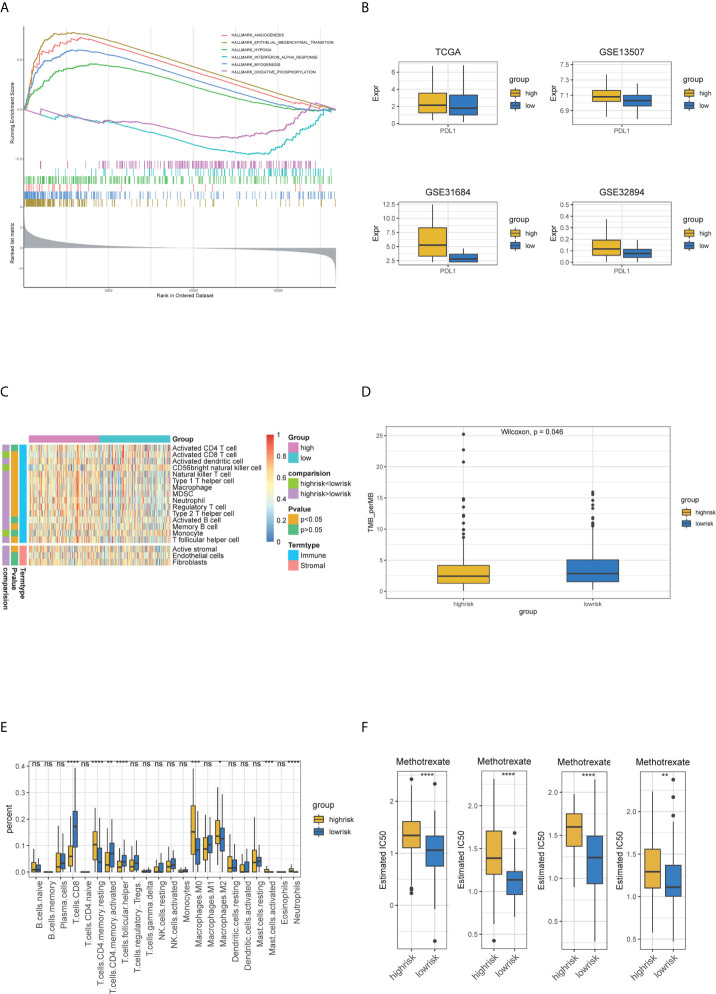
The relationship of five-gene risk groups with functional annotations, TME landscape, immune cell fractions, tumor mutation burden, PDL1 expression and chemotherapy response. **(A)** The Hallmark enrichment of high- and low-risk groups by GSEA method. **(B)** Landscape of immune and stromal microenvironment based on immune and stromal signature. **(C)** Boxplot of the distribution of 22 immune cells in the high- and low-risk groups. **(D)** PDL1 expression differences. **(E)** Tumor mutation burden difference. **(F)** Estimated IC50 indicates the efficiency of chemotherapy to high- and low-risk groups by Methotrexate.

To obtain a comprehensive understanding of the relationship between risk score and the biology of BC, hallmark functional enrichment was performed using GSEA based on the DEGs between high- and low-risk groups. The high-risk groups were enriched in the process of the EMT, angiogenesis, myogenesis and hypoxia, and the low-risk groups were enriched in the process of oxidative phosphorylation and interferon alpha response (**Figure 8A**). The landscape of TME was depicted using enrichment scores calculated based on immune and stromal biomarkers (**Figure 8B**). Low-risk groups had higher enrichment scores in activated CD8+ T cell and CD56 bright natural killer cell, while high-risk groups showed higher enrichment scores in many other immune cells like activated dendritic cell, natural killer T cell and immune suppressive cells such as MDSC and Treg cells (Wilcoxon signed-rank test, *p <*0.05). The high-risk group also had higher enrichment scores in activated stroma, which demonstrated to be associated with poor prognosis (Wilcoxon signed-rank test, *p <*0.05) (17). We then compared the differential infiltration of 22 immune cells between high- and low-risk groups. The results showed that relative to high-risk groups, higher proportion of CD8+ T cells, follicular helper T cells and activated CD4+ memory T cells could be detected in low-risk groups. While resting CD4+ memory T cells, M0/M2 macrophages, activated mast cells and neutrophils showed lower proportions in low-risk groups (Wilcoxon signed-rank test, *p <*0.05) (**Figure 8C**). The high-risk groups had higher PDL1 expressions compared to low-risk groups (Wilcoxon signed-rank test, *p <*0.05) (**Figure 8D**). Gene mutation is an important factor of tumorigenesis and development. We profiled the mutation patterns and found that the high-risk groups had higher frequency of TP53 mutation and low-risk groups had higher frequency of FGFR3 mutations (**Figures S4A, B**). For the CNA status, there was no statistical significance between two groups (**Figure S4C**). Notably, TMB is viewed as a potential biomarker for immunotherapy response and chemotherapy. Compared with high-risk groups, TMB was higher in low-risk groups (Wilcoxon signed-rank test, *p <*0.05) (**Figure 8E**). The different responses for Methotrexate, Vinblastine, Doxorubicin, Cisplatin (MVAC), a combination of drugs often used for BC, between high-risk groups and low-risk groups were calculated based on the GDSC database. The estimated IC50 values showed that low-risk groups had a better response for Methotrexate (Wilcoxon signed-rank test, *p <*0.05) (**Figure 8F**). In summary, the above findings indicated that the five-gene model is associated with different immune cell infiltration, TMB status and drug responses, suggesting that the model maybe helpful in medical decision-making and therapy.

### Screening of the Potential Related Small Candidate Drugs

**Figure 9:**
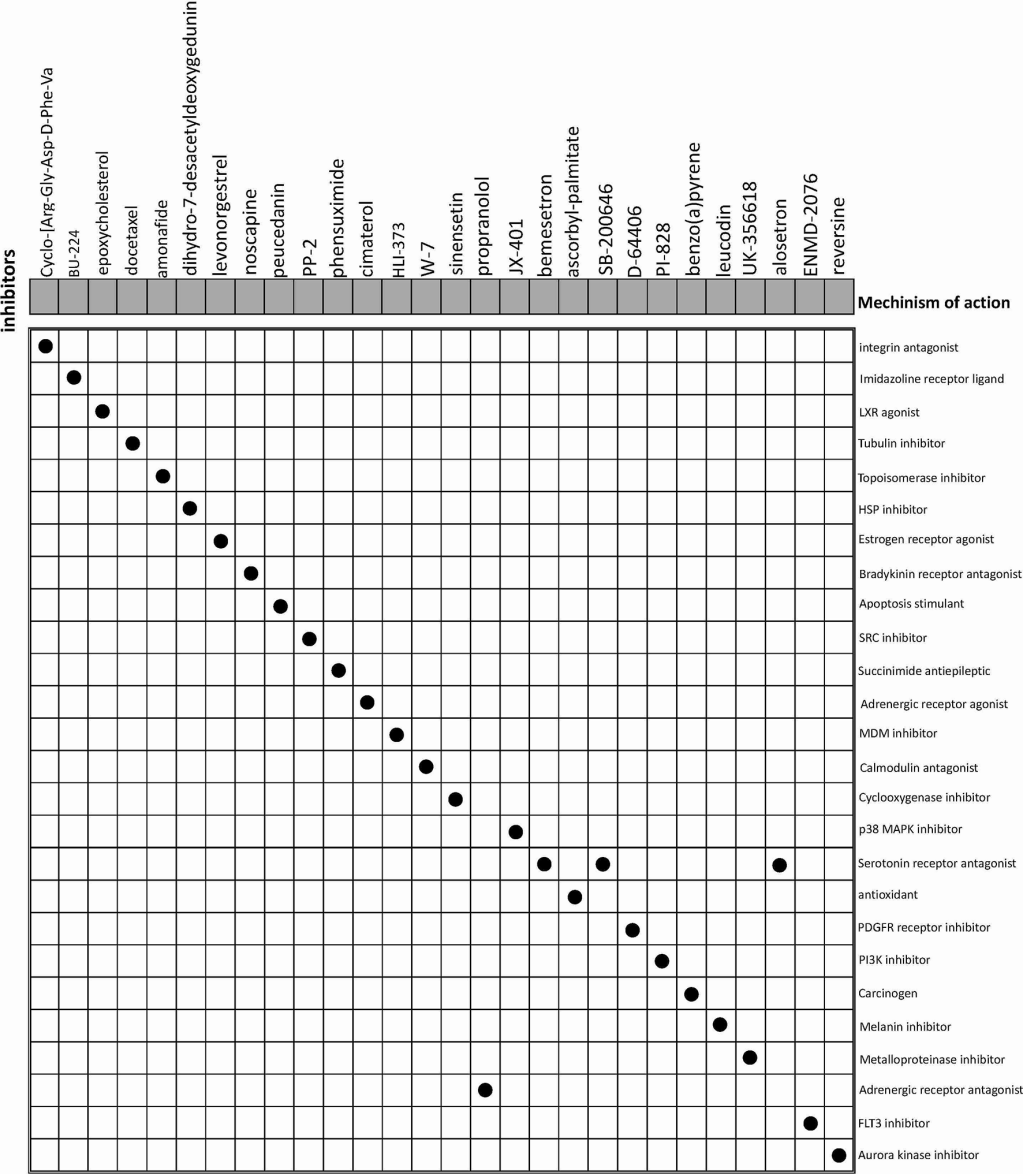
CMap database analysis identifies potential cancidate small molecular drugs targeting the DEGs between high- and low-groups.

The CMap database was employed to seek for potential drugs that capable of using for the treatment of the patients in the high-risk groups. Based on the DEGs between high-risk and low-risk groups, CMap mode-of-action (MoA) analysis indicated mechanisms of action shared by the above inhibitors (**Figure 9**) and small molecule drugs with highly significant correlations (**Table 2**). Positive connectivity scores indicate that drugs can induce biological phenomena in human cell lines. In contrast, negative connectivity scores indicate that the drug reverses the desired biological properties and thus has potential therapeutic value. Docetaxel, Levonorgestrel and Noscapine had been put into clinic and Amonafide was developed in phase 3 trials. These results might provide guidance for future clinical treatment of BC patients.

**Table 2:** Results of CMap analysis (score <−60).

Score	Name	Description	Target
−89.45	BU-224	Imidazoline receptor ligand	MAOA, MAOB
−88.29	Cyclo-[Arg-Gly-Asp-D-Phe-Val]	integrin antagonist	ITGAV, ITGB3
−88.09	epoxycholesterol	LXR agonist	NR1H2, NR1H3
−87.92	docetaxel	Tubulin inhibitor	TUBB, BCL2, MAP2, MAP4, MAPT, NR1I2, TUBB1
−85.06	amonafide	Topoisomerase inhibitor	TOP2A, TOP2B
−81.87	dihydro-7-desacetyldeoxygedunin	HSP inhibitor	HSP90AA1
−78.12	levonorgestrel	Estrogen receptor agonist	PGR, AR, CYP2E1, ESR1, SRD5A1
−68.57	noscapine	Bradykinin receptor antagonist	BDKRB2, SIGMAR1
−67.21	peucedanin	Apoptosis stimulant	
−67.15	PP-2	SRC inhibitor	SRC, LCK, ABL1, LYN, RIPK2
−65.91	phensuximide	Succinimide antiepileptic	
−65.87	cimaterol	Adrenergic receptor agonist	
−65	HLI-373	MDM inhibitor	MDM2
−61.11	W-7	Calmodulin antagonist	CALM1, CALM2, KCNA5, KCNH2, TNNC1, TNNI3

## Discussion

Increasing evidence shows that diverse components of TME, such as immune cells and extracellular matrix, contribute to cancer proliferation, invasion and immune evasion. As the most common malignancy of the urinary system, BC has been frequently studied, but association between TME-related genes and cancer prognosis has not been fully elucidated.

In this study, we calculated the immune and stromal scores of BC samples using the ESTIMATE algorithm and found that the estimated scores were associated with BC progression and prognosis. We characterized the features of the three subclasses divided based on the selected TME-related genes. We found that Class 2, which had a higher fraction of anti-tumor cells such as CD8+ T cells and activated CD4+ memory T cells, had a better prognosis compared to Class 1, which had comparable stromal scores and fractions of immunosuppressive cells, such as Treg cells and M2 macrophages. This result may provide evidence that immune components are associated with BC prognosis. However, the concrete mechanism of how they affect the progression of BC and the interactions between different immune cells need further exploration.

A number of studies have already presented several gene signatures for predicting the prognosis of BC (18–21); however, few studies comprehensively delineated the TME of BC, and there has not been one robust prognosis model based on TME. As we highlighted in the entire study, the TME plays an important role in the development of cancer and affects the growth, invasion, and metastasis of cancer (7, 22–24). It is of great value to explore the prognostic and therapeutic benefits based on TME. In this study, we established a five-gene model based on TME-related genes, and its prognostic value was validated in several independent cohorts. In addition, this model is also a potential predictor of chemosensitivity. It might be useful for clinical medication practice.

Among the members of the five-gene signature, evidence suggests that some of them are associated with the development of BC or other cancers. FPR1, a member of the chemotactic GPCR-7TM formyl peptide receptor family, is expressed on a variety of leukocytes and is primarily involved in leukocyte migration to the sites of bacterial infections. More recently, investigations have revealed that FPR1 plays an important role in cancer. Although the expression of FPR1 in healthy, non-immune cells is low, a number of tumors have been shown to express significant levels of FPR1 (25–30). Importantly, a previous study indicated that targeting FPR1 by ICT12035, a selective small molecule antagonist, can provide a new avenue for the therapy of cancers (31). Therefore, further investigation of FPR1 antagonists can provide opportunities for more efficient treatment of cancers, including bladder cancer. IL10 is a cytokine that has pleiotropic effects on immunoregulation and inflammation. Previous studies reported that IL10 plays an important role in regulating BC immunosurveillance and immunotherapy (19, 32, 33). TNFAIP6 encoded proteins are involved in the EMT process, cell migration, and extracellular matrix stability (34, 35). It is reported to be an inflammation-associated protein regulated by proinflammatory cytokines such as TNFα, interferon-γ, and interleukin-1 (35, 36). It had been demonstrated that high TNFAIP6 expression is significantly associated with aggressive pathological features in urothelial cancer and gastric cancer (37, 38). Incorporating TNFAIP6 immunostaining in current pathological examinations may be useful for identifying high-risk patients to assist in personalized medical decision-making and therapy. GFPT2 encodes for glutamine-fructose-6-phosphate aminotransferase 2, which is responsible for glycosylation. As a rate-limiting enzyme of the hexosamine biosynthesis pathway (HBP), it is involved in human breast and colon tumorigenesis (3, 39). A study confirmed that in lung cancer, normal fibroblasts transformed to cancer-associated fibroblast (CAF)-like cells under TGF-β treatment and upregulated HBP genes which include GFPT2 (40). ZNF683 is known as a transcription factor that mediates a transcriptional program in various innate and adaptive immune tissue-resident lymphocyte T-cell types such as natural killer T cells (41). These studies offered evidences that our selected five-gene signature was indeed related to immune and stromal functions, and some of these genes were demonstrated to be associated with the development of BC and other types of cancers. Thus, our prognostic prediction model composed of evidence-supported genes would be more explainable. We believe that the combination of immunotherapy and FPR1 inhibitors such as Nedocromil and ICT12035, other potential small molecular drugs will be an available approach for clinical trials to further improve the therapeutic efficacy. However, the molecular mechanism by which the five-gene signature affects the prognosis of BC patients requires further experimental work.

Overall, our exploration based on the TME of BC will be beneficial for understanding how the TME components affects the prognosis of BC. The TME-based model may provide useful information for prognostic prediction and guidance for the clinical therapy of BC.

## Data Availability Statement

Publicly available datasets were analyzed in this study. This data can be found here: https://xenabrowsernet/datapages/; https://wwwncbinlmnihgov/geo/query/acccgi?acc=GSE13507; https://www.ncbi.nlm.nih.gov/geo/query/acc.cgi?acc=GSE31684; https://www.ncbi.nlm.nih.gov/geo/query/acc.cgi?acc=GSE32894.

## Author Contributions

The design and data analysis were conducted by XJL. The immumohistochemical staining and drug search results were analyzed by JF. The draft was edited by XL and YS. The project administration was conducted by XL and YS. Review and supervise manuscripts were conducted by XL and YS. All authors contributed to the article and approved the submitted version.

## Funding

This work was supported by grants from the National Natural Science Foundation of China (81872299), Natural Science Foundation of Guangdong Province (2018A0303130090) to XL and China Postdoctoral Science Foundation (2020M683073) to YS.

## Conflict of Interest

The authors declare that the research was conducted in the absence of any commercial or financial relationships that could be construed as a potential conflict of interest.

## Supplementary Material

Supplementary Figure 1 Consensus clustering for BC samples based on TME-related genes. **(A)** Consensus cumulative distribution function plot. **(B)** Consensus matrix for k=3. **(C)** Boxplot of enrichment scores of hallmark features among three subclasses. **(D)** Boxplot of immune suppressive and immune active genes among three subclasses. **(E)** Boxplot of immune checkpoint genes expression among three subclasses. **(F)** Bar plot of the ICB response predictions among three subclasses. **(G)** Forest plot of the log odds ratio for significantly different gene mutations.

Supplementary Figure 2 Risk score analysis, time-dependent ROC analysis and Kaplan–Meier analysis for the validation of prognostic model in **(A)** internal train set and **(B)** internal validation set.

Supplementary Figure 3 Forest plots for clinical factors and risk score. **(A)** Univariate cox forest plot for age, sex, stage and risk score. Age, stage and risk score were statistically significant. **(B)** Multivariate cox forest plot for age, sex, stage and risk score. The risk score calculated by five-gene signature was still an independent predictor. **(C)** The c-index scores for age, stage and riskscore. Time-dependent ROC analysis of age, stage and riskscore for **(D)** one-year survival **(E)** three-year survival **(F)** five-year survival.

Supplementary Figure 4 Mutation and CNV differences of high- and low-risk groups. **(A)** Oncoplot for the top mutation genes. **(B)** Forest plot of the log odds ratio for significantly different gene mutations. **(C)** Numbers of amplification and depletion differences of high- and low-risk groups.
