# Supplementary figures and images for: An Exploration of the Tumor Microenvironment Identified a Novel Five-Gene Model for Predicting Outcomes in Bladder Cancer
Source: Front Oncol. 2021 May 3;11:642527. doi: 10.3389/fonc.2021.642527 (PMC8126988; doi:10.3389/fonc.2021.642527)

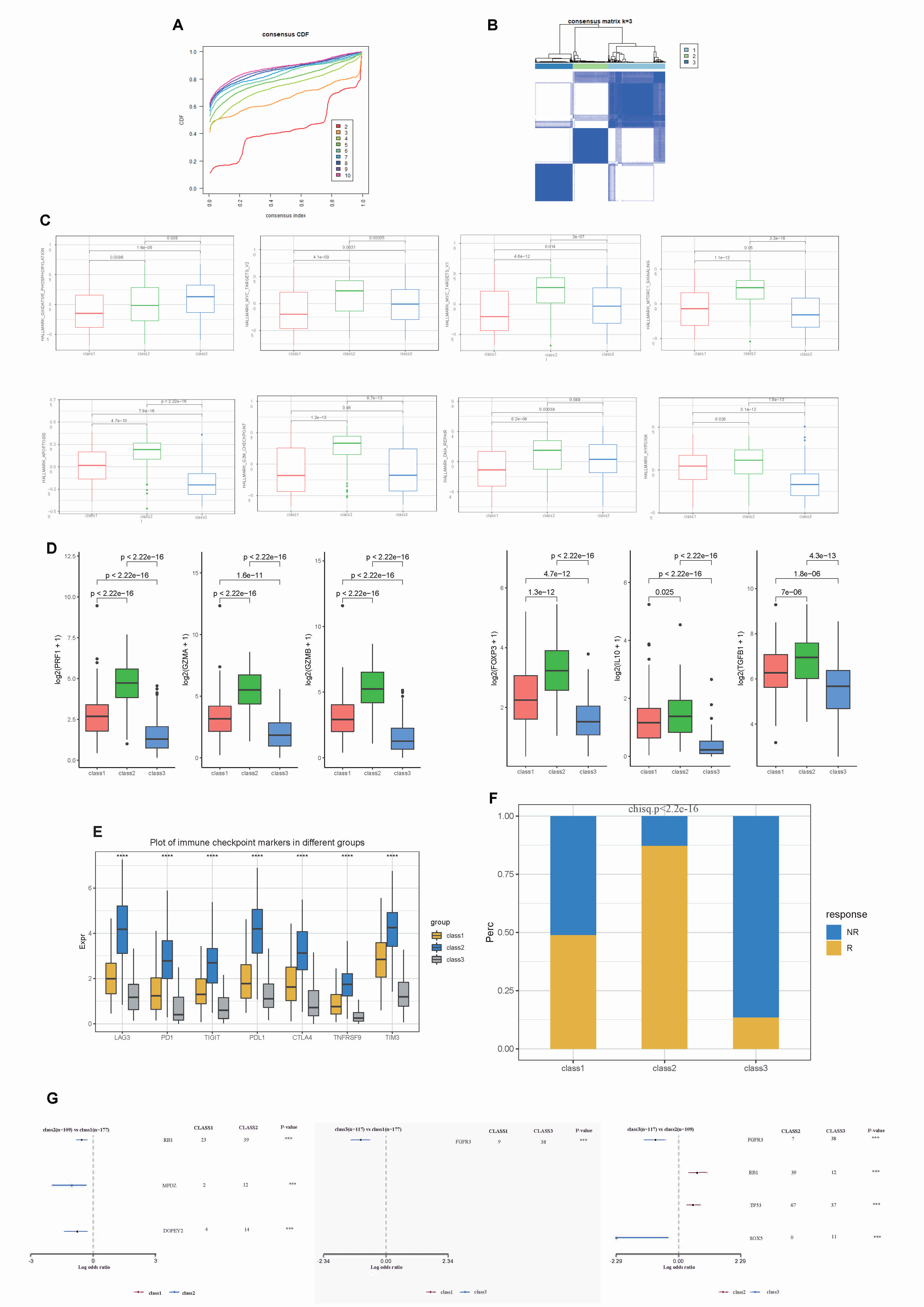

Supplement: Supplementary Figure 1 — Consensus clustering for BC samples based on TME-related genes. (A) Consensus cumulative distribution function plot. (B) Consensus matrix for k=3. (C) Boxplot of enrichment scores of hallmark features among three subclasses. (D) Boxplot of immune suppressive and immune active genes among three subclasses. (E) Boxplot of immune checkpoint genes expression among three subclasses. (F) Bar plot of the ICB response predictions among three subclasses. (G) Forest plot of the log odds ratio for significantly different gene mutations. [file Image_1.tif]

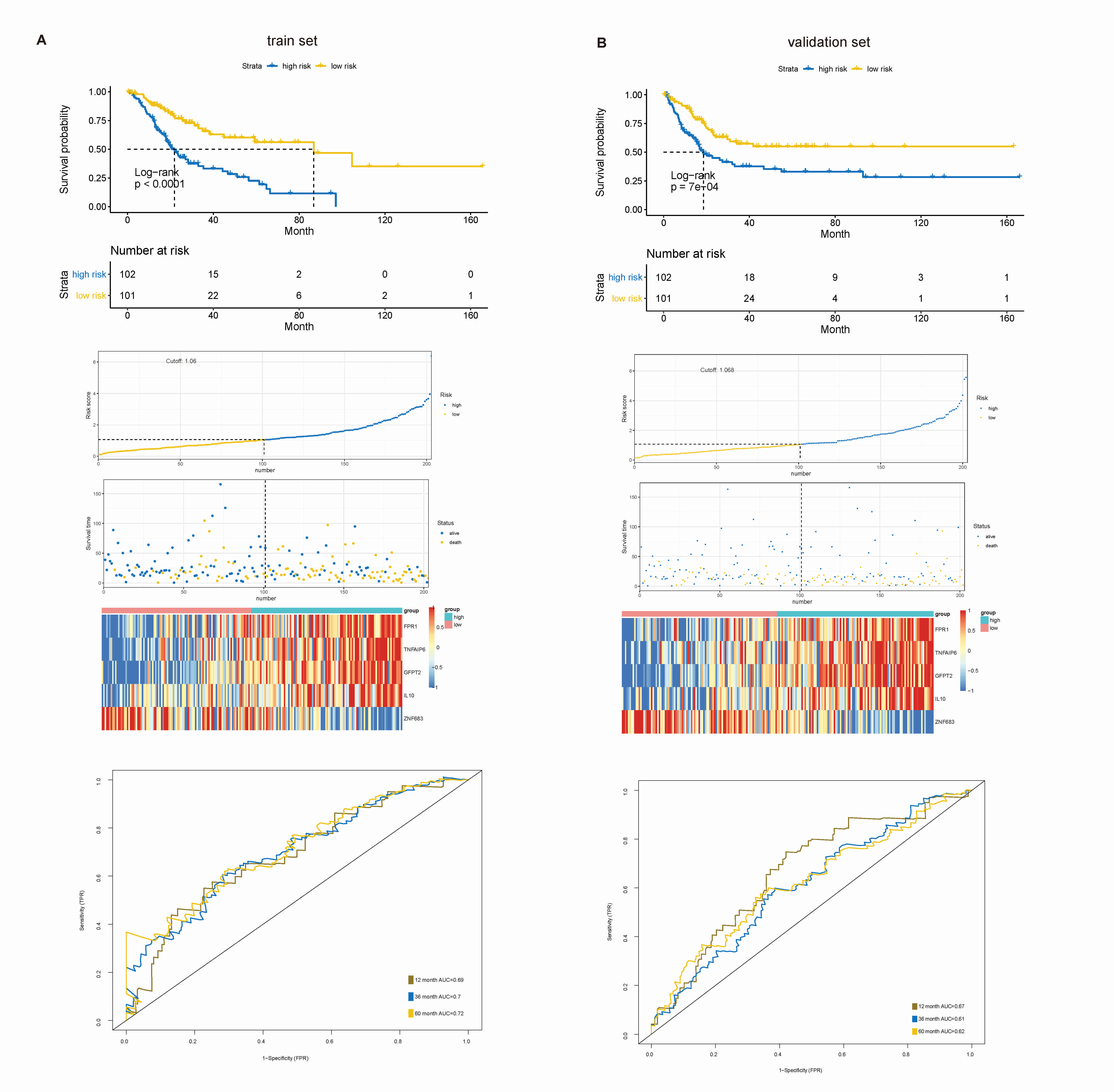

Supplement: Supplementary Figure 2 — Risk score analysis, time-dependent ROC analysis and Kaplan–Meier analysis for the validation of prognostic model in (A) internal train set and (B) internal validation set. [file Image_2.tif]

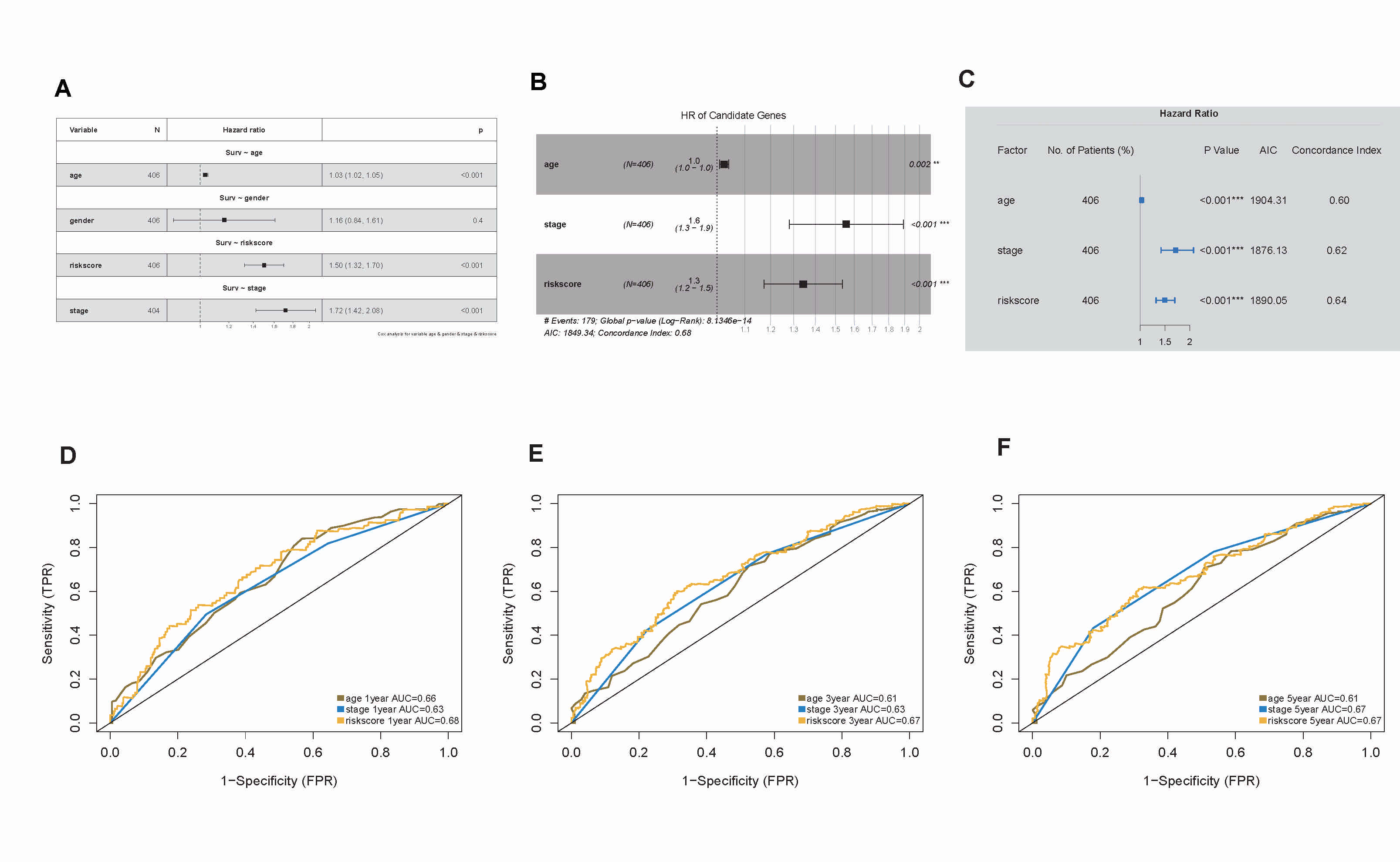

Supplement: Supplementary Figure 3 — Forest plots for clinical factors and risk score. (A) Univariate cox forest plot for age, sex, stage and risk score. Age, stage and risk score were statistically significant. (B) Multivariate cox forest plot for age, sex, stage and risk score. The risk score calculated by five-gene signature was still an independent predictor. (C) The c-index scores for age, stage and riskscore. Time-dependent ROC analysis of age, stage and riskscore for (D) one-year survival (E) three-year survival (F) five-year survival. [file Image_3.tif]

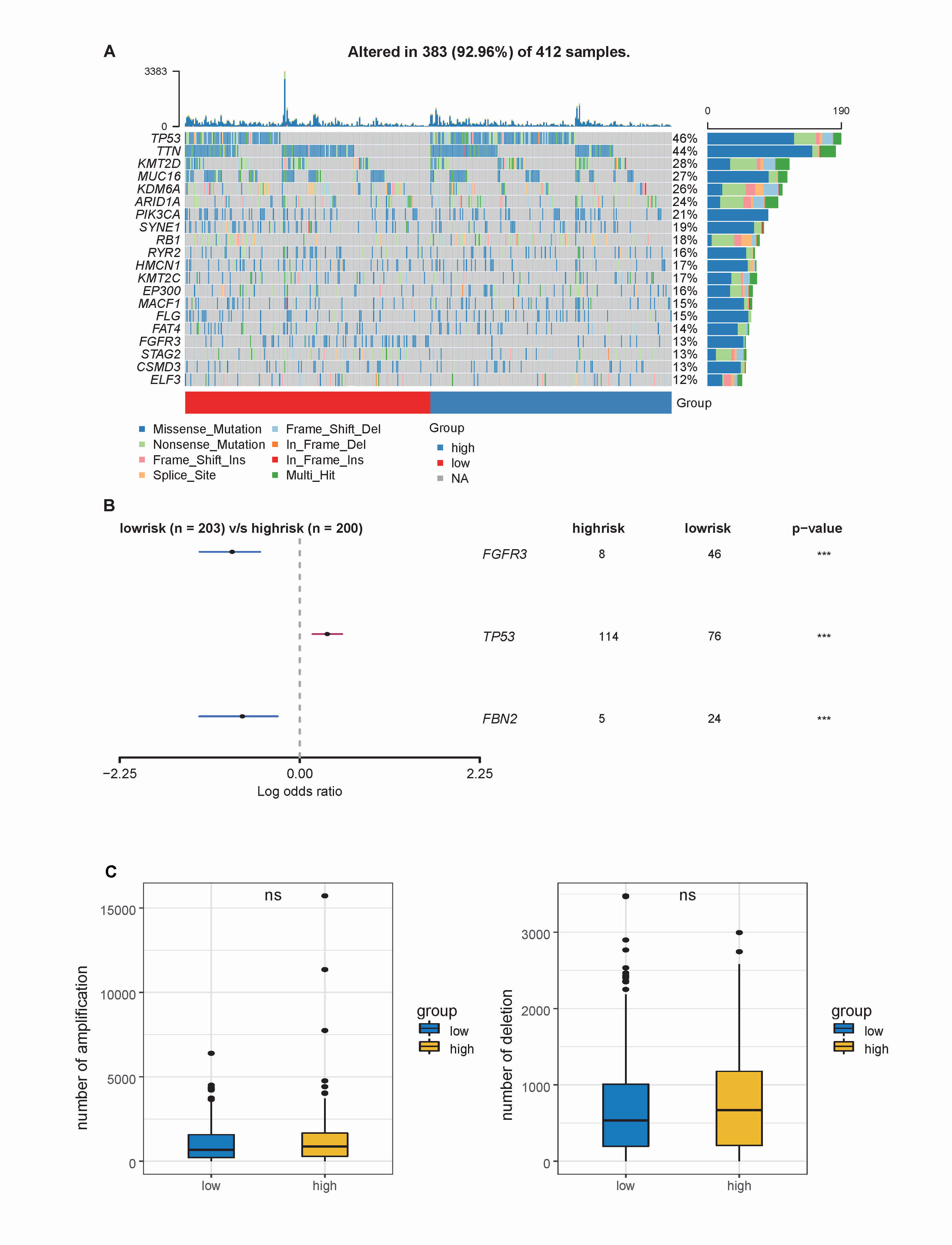

Supplement: Supplementary Figure 4 — Mutation and CNV differences of high- and low-risk groups. (A) Oncoplot for the top mutation genes. (B) Forest plot of the log odds ratio for significantly different gene mutations. (C) Numbers of amplification and depletion differences of high- and low-risk groups. [file Image_4.tif]
