# Supplementary material for: An Exploration of the Tumor Microenvironment Identified a Novel Five-Gene Model for Predicting Outcomes in Bladder Cancer
Source: Front Oncol. 2021 May 3;11:642527. doi: 10.3389/fonc.2021.642527 (PMC8126988; doi:10.3389/fonc.2021.642527)
Supplement: Supplementary file 5 [file Table_1.docx]

TableS1.Univariate Cox hazard ratio regression model of TME-related genes in TCGA BLCA cohort

| gene | HR | Upper | Lower | Pvalue |
| --- | --- | --- | --- | --- |
| FNDC1 | 1.15 | 1.24 | 1.07 | 0.00 |
| ITGA11 | 1.14 | 1.27 | 1.03 | 0.01 |
| CPXM1 | 1.14 | 1.29 | 1.01 | 0.03 |
| AEBP1 | 1.18 | 1.33 | 1.04 | 0.01 |
| ADAMTS2 | 1.16 | 1.31 | 1.03 | 0.02 |
| CILP | 1.10 | 1.17 | 1.03 | 0.01 |
| DCN | 1.16 | 1.31 | 1.02 | 0.02 |
| GLT8D2 | 1.23 | 1.43 | 1.06 | 0.01 |
| LRRC15 | 1.08 | 1.15 | 1.02 | 0.02 |
| OLFML3 | 1.14 | 1.30 | 1.01 | 0.03 |
| COL11A1 | 1.07 | 1.13 | 1.00 | 0.04 |
| COLEC12 | 1.12 | 1.24 | 1.01 | 0.04 |
| FGF7 | 1.16 | 1.26 | 1.06 | 0.00 |
| PDLIM3 | 1.21 | 1.36 | 1.08 | 0.00 |
| CCN2 | 1.14 | 1.30 | 1.01 | 0.04 |
| FPR1 | 1.22 | 1.39 | 1.07 | 0.00 |
| ANXA6 | 1.37 | 1.77 | 1.06 | 0.02 |
| SFRP4 | 1.08 | 1.13 | 1.03 | 0.00 |
| CCDC80 | 1.39 | 1.57 | 1.23 | 0.00 |
| POSTN | 1.12 | 1.21 | 1.03 | 0.01 |
| ISLR | 1.19 | 1.31 | 1.07 | 0.00 |
| DPT | 1.09 | 1.16 | 1.03 | 0.00 |
| GPC6 | 1.15 | 1.27 | 1.04 | 0.01 |
| SFRP2 | 1.09 | 1.15 | 1.04 | 0.00 |
| PLA2G5 | 1.12 | 1.23 | 1.02 | 0.01 |
| FOLR2 | 1.17 | 1.35 | 1.02 | 0.03 |
| COMP | 1.11 | 1.18 | 1.04 | 0.00 |
| COL6A2 | 1.23 | 1.45 | 1.04 | 0.01 |
| BNC2 | 1.20 | 1.34 | 1.08 | 0.00 |
| COL10A1 | 1.08 | 1.14 | 1.02 | 0.01 |
| LYVE1 | 1.12 | 1.25 | 1.01 | 0.04 |
| FAM180A | 1.17 | 1.27 | 1.07 | 0.00 |
| TNFAIP6 | 1.29 | 1.43 | 1.15 | 0.00 |
| F13A1 | 1.18 | 1.32 | 1.06 | 0.00 |
| PLN | 1.10 | 1.18 | 1.03 | 0.01 |
| CNN1 | 1.10 | 1.18 | 1.02 | 0.01 |
| P2RX1 | 1.09 | 1.19 | 1.00 | 0.04 |
| CXCL12 | 1.13 | 1.26 | 1.02 | 0.02 |
| COL6A1 | 1.29 | 1.51 | 1.09 | 0.00 |
| RUBCNL | 1.18 | 1.36 | 1.03 | 0.02 |
| IL10RA | 0.69 | 0.89 | 0.53 | 0.00 |
| LAT2 | 0.75 | 0.97 | 0.58 | 0.03 |
| PRRX1 | 1.20 | 1.33 | 1.08 | 0.00 |
| CPXM2 | 1.15 | 1.27 | 1.04 | 0.00 |
| SYNM | 1.10 | 1.20 | 1.01 | 0.03 |
| HSPB6 | 1.13 | 1.21 | 1.05 | 0.00 |
| CR1 | 1.11 | 1.23 | 1.01 | 0.03 |
| SHISAL1 | 1.16 | 1.27 | 1.06 | 0.00 |
| IGSF21 | 1.12 | 1.23 | 1.02 | 0.02 |
| FAM20C | 1.27 | 1.45 | 1.11 | 0.00 |
| WAS | 0.59 | 0.78 | 0.45 | 0.00 |
| NCF1 | 0.85 | 0.98 | 0.75 | 0.02 |
| CTHRC1 | 1.25 | 1.40 | 1.11 | 0.00 |
| OGN | 1.10 | 1.17 | 1.04 | 0.00 |
| MRO | 1.18 | 1.31 | 1.07 | 0.00 |
| MYO1F | 0.71 | 0.90 | 0.57 | 0.00 |
| HSPB7 | 1.14 | 1.22 | 1.06 | 0.00 |
| GAB3 | 0.67 | 0.86 | 0.52 | 0.00 |
| GREM1 | 1.11 | 1.18 | 1.03 | 0.00 |
| KIAA1755 | 1.22 | 1.35 | 1.10 | 0.00 |
| FLNC | 1.18 | 1.28 | 1.09 | 0.00 |
| CD37 | 0.80 | 0.97 | 0.66 | 0.02 |
| GIMAP4 | 0.75 | 0.92 | 0.61 | 0.01 |
| PARVG | 0.79 | 1.00 | 0.62 | 0.05 |
| CCN4 | 1.20 | 1.32 | 1.08 | 0.00 |
| FAM20A | 1.20 | 1.37 | 1.05 | 0.01 |
| C5AR2 | 1.15 | 1.29 | 1.03 | 0.01 |
| MEOX2 | 1.08 | 1.16 | 1.01 | 0.02 |
| SASH3 | 0.78 | 0.94 | 0.65 | 0.01 |
| LCP2 | 0.72 | 0.90 | 0.58 | 0.00 |
| CEACAM21 | 0.83 | 0.96 | 0.73 | 0.01 |
| GIMAP1 | 0.78 | 0.96 | 0.63 | 0.02 |
| CD4 | 0.77 | 0.91 | 0.65 | 0.00 |
| LST1 | 0.81 | 1.00 | 0.65 | 0.05 |
| ABI3 | 0.77 | 0.94 | 0.62 | 0.01 |
| FERMT3 | 0.74 | 0.93 | 0.59 | 0.01 |
| FAP | 1.19 | 1.33 | 1.07 | 0.00 |
| RSPO3 | 1.08 | 1.17 | 1.01 | 0.03 |
| FHL1 | 1.12 | 1.23 | 1.03 | 0.01 |
| SGCA | 1.08 | 1.16 | 1.01 | 0.03 |
| CD48 | 0.83 | 0.98 | 0.71 | 0.02 |
| HPSE2 | 1.10 | 1.16 | 1.03 | 0.00 |
| HCST | 0.81 | 0.97 | 0.68 | 0.02 |
| PTPRC | 0.86 | 0.99 | 0.75 | 0.04 |
| SCRG1 | 1.10 | 1.16 | 1.03 | 0.00 |
| CASQ2 | 1.11 | 1.17 | 1.05 | 0.00 |
| KLF17 | 1.08 | 1.17 | 1.01 | 0.03 |
| KCNMA1 | 1.18 | 1.32 | 1.05 | 0.00 |
| EMP3 | 1.22 | 1.45 | 1.02 | 0.03 |
| TBXAS1 | 1.27 | 1.50 | 1.07 | 0.01 |
| CD52 | 0.73 | 0.86 | 0.63 | 0.00 |
| GFPT2 | 1.21 | 1.32 | 1.10 | 0.00 |
| PLPP4 | 1.07 | 1.13 | 1.00 | 0.04 |
| IL10 | 1.24 | 1.39 | 1.10 | 0.00 |
| MYO1G | 0.70 | 0.87 | 0.56 | 0.00 |
| LAPTM5 | 0.84 | 0.98 | 0.72 | 0.02 |
| CYTIP | 0.75 | 0.92 | 0.61 | 0.01 |
| TNFSF13B | 0.81 | 0.95 | 0.69 | 0.01 |
| FAM78A | 0.77 | 0.98 | 0.60 | 0.04 |
| ANGPTL1 | 1.10 | 1.19 | 1.02 | 0.02 |
| MMP9 | 1.09 | 1.19 | 1.01 | 0.03 |
| CHRDL2 | 1.11 | 1.18 | 1.05 | 0.00 |
| P2RY13 | 0.88 | 1.00 | 0.78 | 0.05 |
| DES | 1.06 | 1.11 | 1.02 | 0.01 |
| ACTC1 | 1.07 | 1.11 | 1.02 | 0.00 |
| CPA3 | 1.15 | 1.26 | 1.05 | 0.00 |
| CAMK2A | 1.15 | 1.25 | 1.06 | 0.00 |
| CCL5 | 0.87 | 0.97 | 0.79 | 0.01 |
| RFX8 | 1.13 | 1.24 | 1.03 | 0.01 |
| SNX20 | 0.82 | 0.96 | 0.70 | 0.01 |
| CD72 | 0.83 | 0.97 | 0.71 | 0.02 |
| RASSF4 | 0.75 | 0.91 | 0.62 | 0.00 |
| RASAL3 | 0.60 | 0.75 | 0.49 | 0.00 |
| ADRB3 | 1.09 | 1.17 | 1.02 | 0.02 |
| PPP1R16B | 0.78 | 0.93 | 0.66 | 0.00 |
| GALNT17 | 1.12 | 1.21 | 1.04 | 0.00 |
| GNGT2 | 0.81 | 0.96 | 0.68 | 0.01 |
| TPSAB1 | 1.12 | 1.23 | 1.02 | 0.02 |
| MFAP5 | 1.13 | 1.20 | 1.06 | 0.00 |
| PDE4B | 1.28 | 1.50 | 1.08 | 0.00 |
| SLAMF6 | 0.88 | 0.99 | 0.78 | 0.03 |
| CD27 | 0.72 | 0.85 | 0.61 | 0.00 |
| PTGFR | 1.13 | 1.23 | 1.04 | 0.00 |
| LAX1 | 0.89 | 0.99 | 0.80 | 0.04 |
| ARHGAP9 | 0.66 | 0.81 | 0.53 | 0.00 |
| SLAMF1 | 0.86 | 0.98 | 0.76 | 0.02 |
| ARHGAP15 | 0.81 | 0.98 | 0.67 | 0.03 |
| CFP | 0.86 | 0.99 | 0.74 | 0.04 |
| MAP1LC3C | 1.10 | 1.20 | 1.00 | 0.05 |
| JAML | 0.77 | 0.90 | 0.66 | 0.00 |
| CCR5 | 0.78 | 0.89 | 0.68 | 0.00 |
| FGF16 | 1.10 | 1.21 | 1.01 | 0.03 |
| CD5 | 0.82 | 0.97 | 0.69 | 0.02 |
| ICAM3 | 0.84 | 0.94 | 0.75 | 0.00 |
| CORO1A | 0.78 | 0.91 | 0.68 | 0.00 |
| IKZF1 | 0.82 | 0.96 | 0.69 | 0.01 |
| IL21R | 0.86 | 0.98 | 0.76 | 0.02 |
| PTPN7 | 0.73 | 0.87 | 0.61 | 0.00 |
| XPNPEP2 | 1.09 | 1.16 | 1.02 | 0.01 |
| TRAF3IP3 | 0.79 | 0.95 | 0.66 | 0.01 |
| ITK | 0.88 | 0.99 | 0.78 | 0.04 |
| ECRG4 | 1.11 | 1.18 | 1.05 | 0.00 |
| ADCYAP1 | 1.10 | 1.19 | 1.03 | 0.01 |
| LYZ | 0.89 | 0.98 | 0.81 | 0.02 |
| DPEP1 | 1.07 | 1.15 | 1.00 | 0.04 |
| ITGAL | 0.73 | 0.85 | 0.63 | 0.00 |
| ZC3H12D | 0.74 | 0.88 | 0.63 | 0.00 |
| CCIN | 1.13 | 1.23 | 1.03 | 0.01 |
| ROR2 | 1.12 | 1.23 | 1.03 | 0.01 |
| GIMAP5 | 0.87 | 0.98 | 0.77 | 0.02 |
| TMEM255A | 1.14 | 1.25 | 1.03 | 0.01 |
| SPN | 0.76 | 0.88 | 0.65 | 0.00 |
| CHRM2 | 1.06 | 1.12 | 1.01 | 0.02 |
| SPHK1 | 1.15 | 1.28 | 1.04 | 0.01 |
| NIBAN3 | 0.89 | 0.99 | 0.80 | 0.03 |
| SH2D1A | 0.86 | 0.95 | 0.78 | 0.00 |
| CLECL1 | 0.80 | 0.88 | 0.72 | 0.00 |
| TBC1D10C | 0.66 | 0.78 | 0.55 | 0.00 |
| HLA.DPB1 | 0.82 | 0.93 | 0.73 | 0.00 |
| LTA | 0.88 | 0.98 | 0.79 | 0.02 |
| CD3E | 0.76 | 0.87 | 0.67 | 0.00 |
| SIT1 | 0.87 | 0.97 | 0.78 | 0.01 |
| PKDCC | 1.10 | 1.19 | 1.02 | 0.02 |
| PYHIN1 | 0.84 | 0.94 | 0.75 | 0.00 |
| KLHL6 | 0.84 | 0.98 | 0.72 | 0.03 |
| ZNF831 | 0.87 | 0.96 | 0.78 | 0.01 |
| CLMP | 1.11 | 1.22 | 1.00 | 0.05 |
| CXCR3 | 0.87 | 0.96 | 0.78 | 0.01 |
| GRAP2 | 0.84 | 0.98 | 0.72 | 0.02 |
| SERTM2 | 1.08 | 1.15 | 1.01 | 0.02 |
| CD3G | 0.75 | 0.86 | 0.65 | 0.00 |
| HGF | 1.15 | 1.25 | 1.06 | 0.00 |
| AARD | 1.11 | 1.18 | 1.03 | 0.00 |
| MAP4K1 | 0.70 | 0.83 | 0.60 | 0.00 |
| TRAT1 | 0.89 | 0.97 | 0.82 | 0.01 |
| PRF1 | 0.87 | 0.98 | 0.78 | 0.02 |
| ASB5 | 1.05 | 1.10 | 1.00 | 0.03 |
| ITGA4 | 1.16 | 1.32 | 1.02 | 0.02 |
| SAMD3 | 0.86 | 0.97 | 0.76 | 0.02 |
| HLA.DMB | 0.80 | 0.92 | 0.69 | 0.00 |
| SLA2 | 0.79 | 0.90 | 0.70 | 0.00 |
| CD2 | 0.82 | 0.92 | 0.73 | 0.00 |
| BPI | 1.16 | 1.27 | 1.06 | 0.00 |
| TNFAIP8L3 | 1.24 | 1.38 | 1.12 | 0.00 |
| ICOS | 0.91 | 0.99 | 0.83 | 0.03 |
| HLA.DQA1 | 0.87 | 0.97 | 0.78 | 0.01 |
| RSPO2 | 1.08 | 1.14 | 1.02 | 0.01 |
| C4orf54 | 1.09 | 1.16 | 1.02 | 0.01 |
| GPR68 | 1.12 | 1.26 | 1.00 | 0.04 |
| IL12RB1 | 0.84 | 0.95 | 0.73 | 0.01 |
| C7 | 1.06 | 1.11 | 1.01 | 0.02 |
| SP140 | 0.83 | 0.94 | 0.73 | 0.00 |
| MSC | 1.15 | 1.28 | 1.04 | 0.01 |
| CD6 | 0.82 | 0.96 | 0.71 | 0.01 |
| NKG7 | 0.82 | 0.91 | 0.73 | 0.00 |
| SCN2B | 1.09 | 1.17 | 1.01 | 0.03 |
| SYNDIG1 | 1.08 | 1.15 | 1.01 | 0.03 |
| GZMM | 0.83 | 0.94 | 0.73 | 0.00 |
| IL32 | 0.81 | 0.91 | 0.72 | 0.00 |
| GZMH | 0.88 | 0.96 | 0.81 | 0.00 |
| FAIM2 | 1.14 | 1.24 | 1.05 | 0.00 |
| C14orf180 | 1.08 | 1.16 | 1.00 | 0.04 |
| TPSD1 | 1.07 | 1.14 | 1.01 | 0.03 |
| ARSI | 1.17 | 1.27 | 1.08 | 0.00 |
| BTLA | 0.84 | 0.92 | 0.76 | 0.00 |
| TIGIT | 0.82 | 0.93 | 0.73 | 0.00 |
| GPR18 | 0.81 | 0.92 | 0.72 | 0.00 |
| SSTR3 | 0.91 | 0.99 | 0.83 | 0.03 |
| NUGGC | 0.89 | 0.98 | 0.80 | 0.02 |
| CTLA4 | 0.90 | 0.99 | 0.82 | 0.03 |
| CHRDL1 | 1.09 | 1.14 | 1.03 | 0.00 |
| UBASH3A | 0.84 | 0.94 | 0.76 | 0.00 |
| LAG3 | 0.85 | 0.95 | 0.77 | 0.00 |
| LTB | 0.87 | 0.99 | 0.77 | 0.03 |
| SFRP1 | 1.07 | 1.14 | 1.01 | 0.02 |
| TBX21 | 0.82 | 0.91 | 0.73 | 0.00 |
| PTX3 | 1.07 | 1.15 | 1.00 | 0.04 |
| CALHM6 | 0.88 | 0.97 | 0.79 | 0.01 |
| ANKRD29 | 1.14 | 1.24 | 1.05 | 0.00 |
| GPR1 | 1.13 | 1.22 | 1.06 | 0.00 |
| CD7 | 0.87 | 0.96 | 0.79 | 0.00 |
| SRPX | 1.17 | 1.29 | 1.07 | 0.00 |
| PDCD1 | 0.90 | 0.98 | 0.82 | 0.02 |
| GPR171 | 0.86 | 0.95 | 0.79 | 0.00 |
| TNC | 1.15 | 1.26 | 1.04 | 0.00 |
| IL2RG | 0.80 | 0.91 | 0.70 | 0.00 |
| BFSP2 | 0.90 | 0.99 | 0.81 | 0.03 |
| HLA.DRB1 | 0.88 | 0.98 | 0.80 | 0.02 |
| FGF5 | 1.06 | 1.13 | 1.00 | 0.04 |
| CES1 | 1.11 | 1.16 | 1.05 | 0.00 |
| GZMB | 0.90 | 0.98 | 0.83 | 0.01 |
| F2RL2 | 1.19 | 1.29 | 1.10 | 0.00 |
| HLA.DQB1 | 0.88 | 0.97 | 0.79 | 0.01 |
| CD40LG | 0.90 | 0.99 | 0.82 | 0.03 |
| PENK | 1.08 | 1.17 | 1.01 | 0.03 |
| IL27 | 0.89 | 0.97 | 0.80 | 0.01 |
| GZMA | 0.89 | 0.97 | 0.82 | 0.01 |
| CD8A | 0.86 | 0.95 | 0.78 | 0.00 |
| SPTA1 | 1.08 | 1.17 | 1.00 | 0.04 |
| ADGRG5 | 0.88 | 0.96 | 0.80 | 0.00 |
| SCML4 | 0.86 | 0.94 | 0.78 | 0.00 |
| THEMIS | 0.90 | 0.98 | 0.83 | 0.02 |
| ZNF80 | 0.89 | 0.98 | 0.81 | 0.02 |
| ACOD1 | 0.90 | 0.99 | 0.81 | 0.04 |
| ZNF683 | 0.87 | 0.93 | 0.81 | 0.00 |
| GBP5 | 0.92 | 1.00 | 0.85 | 0.05 |
| TLL1 | 1.16 | 1.24 | 1.07 | 0.00 |
| GABRP | 1.04 | 1.08 | 1.00 | 0.04 |
| IGLL1 | 0.87 | 0.97 | 0.79 | 0.01 |
| SOST | 1.06 | 1.12 | 1.01 | 0.02 |
| APCDD1L | 1.12 | 1.19 | 1.06 | 0.00 |
| CXCL10 | 0.94 | 1.00 | 0.88 | 0.05 |
| GTSF1L | 0.84 | 0.96 | 0.74 | 0.01 |
| KLK5 | 1.04 | 1.08 | 1.01 | 0.01 |
| CXCL11 | 0.94 | 1.00 | 0.89 | 0.04 |
| HMHB1 | 0.87 | 0.98 | 0.77 | 0.03 |
| NEFL | 1.07 | 1.12 | 1.02 | 0.00 |
| FCRL4 | 0.89 | 0.98 | 0.80 | 0.02 |
| KIR2DL3 | 0.91 | 1.00 | 0.82 | 0.05 |
| IL21 | 0.80 | 0.95 | 0.68 | 0.01 |
| S100A7 | 1.03 | 1.06 | 1.00 | 0.04 |
| RPE65 | 1.07 | 1.13 | 1.01 | 0.02 |
| SPANXB1 | 1.06 | 1.12 | 1.00 | 0.04 |
| SPINK4 | 0.95 | 0.99 | 0.91 | 0.02 |
| CRTAC1 | 0.95 | 0.99 | 0.92 | 0.01 |
| ATOH8 | 0.94 | 0.99 | 0.90 | 0.02 |
| KCTD16 | 0.92 | 0.98 | 0.87 | 0.01 |
| TMEM178A | 0.94 | 0.99 | 0.88 | 0.03 |
| REG4 | 0.93 | 0.97 | 0.88 | 0.00 |
| SLC26A5 | 0.91 | 0.98 | 0.86 | 0.01 |
| BPIFA2 | 0.91 | 0.97 | 0.86 | 0.00 |
| UGT2B28 | 0.94 | 0.99 | 0.90 | 0.01 |
| GRM3 | 0.94 | 0.99 | 0.89 | 0.03 |
| UGT2B15 | 0.94 | 0.98 | 0.90 | 0.00 |
| BRINP2 | 0.94 | 1.00 | 0.89 | 0.04 |
| KRTAP5.10 | 0.93 | 0.98 | 0.89 | 0.01 |
| LGALS4 | 0.94 | 0.98 | 0.90 | 0.00 |
| UGT2B11 | 0.90 | 0.97 | 0.84 | 0.00 |
| BTBD16 | 0.97 | 1.00 | 0.94 | 0.05 |
| CYP4F8 | 0.96 | 0.99 | 0.93 | 0.00 |
| MPPED1 | 0.90 | 0.97 | 0.83 | 0.00 |
| ERN2 | 0.96 | 1.00 | 0.93 | 0.04 |
| GLP1R | 0.94 | 1.00 | 0.89 | 0.05 |
| A1CF | 0.91 | 0.97 | 0.85 | 0.01 |
| CYP2C9 | 0.95 | 0.99 | 0.90 | 0.02 |
| UGT1A1 | 0.95 | 0.99 | 0.92 | 0.02 |
| SERPINA7 | 0.92 | 0.99 | 0.86 | 0.03 |
| SLC17A4 | 0.89 | 0.97 | 0.81 | 0.01 |
| DPP10 | 0.95 | 1.00 | 0.90 | 0.04 |
| CLDN6 | 1.07 | 1.12 | 1.01 | 0.02 |
| TFAP2B | 0.95 | 0.99 | 0.91 | 0.02 |
| BHMT | 0.96 | 0.99 | 0.93 | 0.01 |
| UGT2B10 | 0.87 | 0.98 | 0.78 | 0.02 |
| AADACL2 | 1.08 | 1.14 | 1.03 | 0.00 |
| GC | 0.95 | 1.00 | 0.90 | 0.05 |
| PHGR1 | 0.96 | 0.99 | 0.92 | 0.02 |
| PAGE4 | 1.08 | 1.15 | 1.01 | 0.03 |
| SLC17A1 | 0.86 | 0.98 | 0.75 | 0.02 |
| AL583836.1 | 0.87 | 0.99 | 0.76 | 0.04 |
| RHOXF2B | 1.10 | 1.21 | 1.00 | 0.04 |
